# 1236. Update on the *In Vitro* Activity of Ceftaroline against *Staphylococcus aureus* from United States (US) Medical Centers Stratified by Infection Type (2018-2020)

**DOI:** 10.1093/ofid/ofab466.1428

**Published:** 2021-12-04

**Authors:** Helio S Sader, Mariana Castanheira, Mariana Castanheira, Leonard R Duncan, Rodrigo E Mendes

**Affiliations:** JMI Laboratories, North Liberty, Iowa

## Abstract

**Background:**

Ceftaroline was initially approved by the US FDA in 2010 to treat skin and skin structure infection (SSSI) and community-acquired bacterial pneumonia (CABP). FDA approval was extended in 2015 to treat patients with SSSI and CABP who developed bacteremia. Moreover, ceftaroline has also been used off-label to treat other infection types. We evaluated the *in vitro* activity of ceftaroline against *S. aureus* isolated in US medical centers in 2018-2020.

**Methods:**

A total of 9,268 *S. aureus* isolates were consecutively collected from 33 US medical centers in 2018-2020 and susceptibility tested by broth microdilution method against ceftaroline and comparators. Results were stratified by infection type and resistance profile.

**Results:**

Ceftaroline (MIC_50/90_, 0.25/1 mg/L) susceptibility (S) ranged from 98.5% (SSSI) to 95.4% (pneumonia; 97.2% overall [Table]). Ceftaroline retained potent activity and broad spectrum against methicillin-resistant *S. aureus* (MRSA; 41.9% of isolates), with S rates varying from 96.3% (SSSI) to 89.2% (pneumonia; 93.4% overall). Overall S rate to erythromycin (ERY), levofloxacin (LEV), tetracycline (TET), and trimethoprim-sulfamethoxazole (TMP-SMX) were 44.0%, 67.9%, 94.1%, and 97.5%, respectively. Ceftaroline retained good activity against *S. aureus* resistant to ERY (94.8%S), LEV (91.4%S), TET (92.3%S), and/or TMP-SMX (98.7%S). Among the resistant subsets, ceftaroline S rates were generally highest among isolates from SSSI (93.1-100.0%), followed by other infections (81.8-100.0%), bloodstream infections (BSI; 89.4-96.2%), and pneumonia (86.6-98.1%); overall susceptibility was highest among TMP-SMX-R isolates (98.7%), followed by ERY-R (94.8%), MRSA (93.4%), TET-R (92.3%), and LEV-R (91.4%) isolates. Dalbavancin (MIC_90_, 0.03 mg/L), teicoplanin (MIC_90_, 0.5 mg/L), and vancomycin (MIC_90_, 1 mg/L) exhibited complete activity (100.0%S), whereas daptomycin (MIC_90_, 0.5 mg/L) and linezolid (MIC_90_, 2 mg/L) were active against >99.9% of isolates.

**Conclusion:**

Ceftaroline remained very active against contemporary (2018-2020) *S. aureus* from US medical centers, independent of infection type. Ceftaroline retained good activity against MRSA and isolates resistant to ERY, LEV, TET, and/or TMP-SMX.

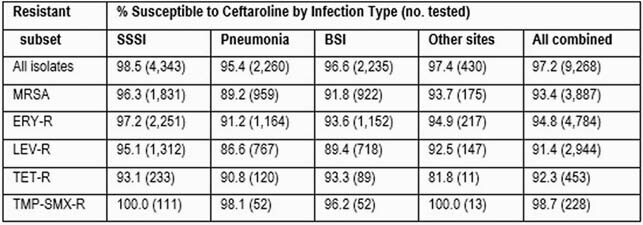

**Disclosures:**

**Helio S. Sader, MD, PhD, FIDSA**, **AbbVie (formerly Allergan**) (Research Grant or Support)**Basilea Pharmaceutica International, Ltd.** (Research Grant or Support)**Cipla Therapeutics** (Research Grant or Support)**Cipla USA Inc.** (Research Grant or Support)**Department of Health and Human Services** (Research Grant or Support, Contract no. HHSO100201600002C)**Melinta Therapeutics, LLC** (Research Grant or Support)**Nabriva Therapeutics** (Research Grant or Support)**Pfizer, Inc.** (Research Grant or Support)**Shionogi** (Research Grant or Support)**Spero Therapeutics** (Research Grant or Support) **Mariana Castanheira, PhD**, **AbbVie (formerly Allergan**) (Research Grant or Support)**Bravos Biosciences** (Research Grant or Support)**Cidara Therapeutics, Inc.** (Research Grant or Support)**Cipla Therapeutics** (Research Grant or Support)**Cipla USA Inc.** (Research Grant or Support)**GlaxoSmithKline** (Research Grant or Support)**Melinta Therapeutics, Inc.** (Research Grant or Support)**Melinta Therapeutics, LLC** (Research Grant or Support)**Pfizer, Inc.** (Research Grant or Support)**Qpex Biopharma** (Research Grant or Support)**Shionogi** (Research Grant or Support)**Spero Therapeutics** (Research Grant or Support) **Mariana Castanheira, PhD**, Affinity Biosensors (Individual(s) Involved: Self): Research Grant or Support; Allergan (Individual(s) Involved: Self): Research Grant or Support; Amicrobe, Inc (Individual(s) Involved: Self): Research Grant or Support; Amplyx Pharma (Individual(s) Involved: Self): Research Grant or Support; Artugen Therapeutics USA, Inc. (Individual(s) Involved: Self): Research Grant or Support; Astellas (Individual(s) Involved: Self): Research Grant or Support; Basilea (Individual(s) Involved: Self): Research Grant or Support; Beth Israel Deaconess Medical Center (Individual(s) Involved: Self): Research Grant or Support; BIDMC (Individual(s) Involved: Self): Research Grant or Support; bioMerieux Inc. (Individual(s) Involved: Self): Research Grant or Support; BioVersys Ag (Individual(s) Involved: Self): Research Grant or Support; Bugworks (Individual(s) Involved: Self): Research Grant or Support; Cidara (Individual(s) Involved: Self): Research Grant or Support; Cipla (Individual(s) Involved: Self): Research Grant or Support; Contrafect (Individual(s) Involved: Self): Research Grant or Support; Cormedix (Individual(s) Involved: Self): Research Grant or Support; Crestone, Inc. (Individual(s) Involved: Self): Research Grant or Support; Curza (Individual(s) Involved: Self): Research Grant or Support; CXC7 (Individual(s) Involved: Self): Research Grant or Support; Entasis (Individual(s) Involved: Self): Research Grant or Support; Fedora Pharmaceutical (Individual(s) Involved: Self): Research Grant or Support; Fimbrion Therapeutics (Individual(s) Involved: Self): Research Grant or Support; Fox Chase (Individual(s) Involved: Self): Research Grant or Support; GlaxoSmithKline (Individual(s) Involved: Self): Research Grant or Support; Guardian Therapeutics (Individual(s) Involved: Self): Research Grant or Support; Hardy Diagnostics (Individual(s) Involved: Self): Research Grant or Support; IHMA (Individual(s) Involved: Self): Research Grant or Support; Janssen Research & Development (Individual(s) Involved: Self): Research Grant or Support; Johnson & Johnson (Individual(s) Involved: Self): Research Grant or Support; Kaleido Biosceinces (Individual(s) Involved: Self): Research Grant or Support; KBP Biosciences (Individual(s) Involved: Self): Research Grant or Support; Luminex (Individual(s) Involved: Self): Research Grant or Support; Matrivax (Individual(s) Involved: Self): Research Grant or Support; Mayo Clinic (Individual(s) Involved: Self): Research Grant or Support; Medpace (Individual(s) Involved: Self): Research Grant or Support; Meiji Seika Pharma Co., Ltd. (Individual(s) Involved: Self): Research Grant or Support; Melinta (Individual(s) Involved: Self): Research Grant or Support; Menarini (Individual(s) Involved: Self): Research Grant or Support; Merck (Individual(s) Involved: Self): Research Grant or Support; Meridian Bioscience Inc. (Individual(s) Involved: Self): Research Grant or Support; Micromyx (Individual(s) Involved: Self): Research Grant or Support; MicuRx (Individual(s) Involved: Self): Research Grant or Support; N8 Medical (Individual(s) Involved: Self): Research Grant or Support; Nabriva (Individual(s) Involved: Self): Research Grant or Support; National Institutes of Health (Individual(s) Involved: Self): Research Grant or Support; National University of Singapore (Individual(s) Involved: Self): Research Grant or Support; North Bristol NHS Trust (Individual(s) Involved: Self): Research Grant or Support; Novome Biotechnologies (Individual(s) Involved: Self): Research Grant or Support; Paratek (Individual(s) Involved: Self): Research Grant or Support; Pfizer (Individual(s) Involved: Self): Research Grant or Support; Prokaryotics Inc. (Individual(s) Involved: Self): Research Grant or Support; QPEX Biopharma (Individual(s) Involved: Self): Research Grant or Support; Rhode Island Hospital (Individual(s) Involved: Self): Research Grant or Support; RIHML (Individual(s) Involved: Self): Research Grant or Support; Roche (Individual(s) Involved: Self): Research Grant or Support; Roivant (Individual(s) Involved: Self): Research Grant or Support; Salvat (Individual(s) Involved: Self): Research Grant or Support; Scynexis (Individual(s) Involved: Self): Research Grant or Support; SeLux Diagnostics (Individual(s) Involved: Self): Research Grant or Support; Shionogi (Individual(s) Involved: Self): Research Grant or Support; Specific Diagnostics (Individual(s) Involved: Self): Research Grant or Support; Spero (Individual(s) Involved: Self): Research Grant or Support; SuperTrans Medical LT (Individual(s) Involved: Self): Research Grant or Support; T2 Biosystems (Individual(s) Involved: Self): Research Grant or Support; The University of Queensland (Individual(s) Involved: Self): Research Grant or Support; Thermo Fisher Scientific (Individual(s) Involved: Self): Research Grant or Support; Tufts Medical Center (Individual(s) Involved: Self): Research Grant or Support; Universite de Sherbrooke (Individual(s) Involved: Self): Research Grant or Support; University of Iowa (Individual(s) Involved: Self): Research Grant or Support; University of Iowa Hospitals and Clinics (Individual(s) Involved: Self): Research Grant or Support; University of Wisconsin (Individual(s) Involved: Self): Research Grant or Support; UNT System College of Pharmacy (Individual(s) Involved: Self): Research Grant or Support; URMC (Individual(s) Involved: Self): Research Grant or Support; UT Southwestern (Individual(s) Involved: Self): Research Grant or Support; VenatoRx (Individual(s) Involved: Self): Research Grant or Support; Viosera Therapeutics (Individual(s) Involved: Self): Research Grant or Support; Wayne State University (Individual(s) Involved: Self): Research Grant or Support **Leonard R. Duncan, PhD**, **AbbVie (formerly Allergan**) (Research Grant or Support)**Basilea Pharmaceutica International, Ltd.** (Research Grant or Support)**Cipla Therapeutics** (Research Grant or Support)**Cipla USA Inc.** (Research Grant or Support)**Department of Health and Human Services** (Research Grant or Support, Contract no. HHSO100201600002C)**Shionogi** (Research Grant or Support) **Rodrigo E. Mendes, PhD**, **AbbVie** (Research Grant or Support)**AbbVie (formerly Allergan**) (Research Grant or Support)**Cipla Therapeutics** (Research Grant or Support)**Cipla USA Inc.** (Research Grant or Support)**ContraFect Corporation** (Research Grant or Support)**GlaxoSmithKline, LLC** (Research Grant or Support)**Melinta Therapeutics, Inc.** (Research Grant or Support)**Melinta Therapeutics, LLC** (Research Grant or Support)**Nabriva Therapeutics** (Research Grant or Support)**Pfizer, Inc.** (Research Grant or Support)**Shionogi** (Research Grant or Support)**Spero Therapeutics** (Research Grant or Support)

